# Applying Image-Based Food-Recognition Systems on Dietary Assessment: A Systematic Review

**DOI:** 10.1093/advances/nmac078

**Published:** 2022-07-08

**Authors:** Kalliopi V Dalakleidi, Marina Papadelli, Ioannis Kapolos, Konstantinos Papadimitriou

**Affiliations:** Department of Food Science and Technology, University of the Peloponnese, Kalamata, Greece; Department of Food Science and Technology, University of the Peloponnese, Kalamata, Greece; Department of Food Science and Technology, University of the Peloponnese, Kalamata, Greece; Laboratory of Food Quality Control and Hygiene, Department of Food Science and Human Nutrition, Agricultural University of Athens, Athens, Greece

**Keywords:** nutrition monitoring, food image recognition, dietary assessment, machine learning, deep learning, artificial intelligence, computer vision, image-based food recognition

## Abstract

Dietary assessment can be crucial for the overall well-being of humans and, at least in some instances, for the prevention and management of chronic, life-threatening diseases. Recall and manual record-keeping methods for food-intake monitoring are available, but often inaccurate when applied for a long period of time. On the other hand, automatic record-keeping approaches that adopt mobile cameras and computer vision methods seem to simplify the process and can improve current human-centric diet-monitoring methods. Here we present an extended critical literature overview of image-based food-recognition systems (IBFRS) combining a camera of the user's mobile device with computer vision methods and publicly available food datasets (PAFDs). In brief, such systems consist of several phases, such as the segmentation of the food items on the plate, the classification of the food items in a specific food category, and the estimation phase of volume, calories, or nutrients of each food item. A total of 159 studies were screened in this systematic review of IBFRS. A detailed overview of the methods adopted in each of the 78 included studies of this systematic review of IBFRS is provided along with their performance on PAFDs. Studies that included IBFRS without presenting their performance in at least 1 of the above-mentioned phases were excluded. Among the included studies, 45 (58%) studies adopted deep learning methods and especially convolutional neural networks (CNNs) in at least 1 phase of the IBFRS with input PAFDs. Among the implemented techniques, CNNs outperform all other approaches on the PAFDs with a large volume of data, since the richness of these datasets provides adequate training resources for such algorithms. We also present evidence for the benefits of application of IBFRS in professional dietetic practice. Furthermore, challenges related to the IBFRS presented here are also thoroughly discussed along with future directions.

## Teaser

**Statement of Significance**: The latest advances of computer vision approaches for dietary assessment are described in this review, and recent applications of image-based food recognition systems (IBFRS) in professional dietetic practice are presented. Open issues that should be tackled in the near future via interdisciplinary research to optimize the performance of IBFRS as well as to increase their adoption by the professionals of the field have been examined and discussed.

## Introduction

Deviations from a healthy and balanced diet may hinder our wellness and lead to chronic and life-threatening diseases (1–4). To ensure a healthy and balanced diet, nutritionists or medical personnel often ask people to keep a manual record or recall the consumed meals and drinks daily. Recall or manual record-keeping methods consist of 3 main approaches: food records, 24-h dietary recall, and food-frequency questionnaires (FFQs) (5). Food records are based on notes of the individual during or after each consumed meal for a specific period of time. A 24-h dietary recall is based on the oral and written data that the individual provides to the medical professional/nutritionist/caregiver regarding the type and amount of consumed food during the previous day. FFQs are recall methods that store the frequency or portion size about food and beverage consumption over a long period of time, such as a month or a year. All methods of recall and manual record keeping are simple to follow and costless, but are tedious and the individuals often fail to comply for the whole period of the recording time (5) or often underestimate the quantity of consumed food/drinks up to 33% (6). In addition, individuals might fail to remember or even deliberately not record all details regarding their meals (6). Thus, methods for automatic record keeping can play a fundamental role in making dietary habits monitoring more objective and accurate.

Researchers used pictures of common foods in different portion sizes for aiding users to fill in paper-based FFQs back in the 1980s (7). Recent approaches for diet monitoring that are based on mobile applications [MyFitnessPal, See How You Eat, MyPlate, Protein Tracker, Fooducate (8)] have been adopted by many researchers and practicing dietitians (9). Such mobile applications use input from the user in the form of images, dropdown menus, and text, and have shown great potential for aiding individuals in recording and improving their dietary habits, since the user is informed about the calorific content of the meal consumed and suggestions are provided about next meals for a healthy and balanced diet. However, such applications require a significant amount of input from the user; thus, they have similar drawbacks as recall and manual record-keeping approaches.

To improve dietary intake monitoring, several automatic record keeping approaches have been proposed. Such methods are based on gesture recognition, chewing and swallowing recognition, and camera-based methods (10). Although sensors for gesture, chewing, and swallowing recognition are easy to wear, they can provide only general information about the consumed meal; thus, they can be useful only as additional, secondary information for food recognition (11).

Camera-based methods use current mobile imaging technologies for dietary intake monitoring. To maximize the automation of the procedure of food record keeping the use of computer vision and machine-learning methods has been suggested (12). The flow of the dietary assessment systems combining a camera of the user's mobile device with computer vision methods, also called image-based food recognition systems (IBFRS), is as follows: *1*) the user takes a photograph of the upcoming meal with a camera of his/her mobile device, *2*) the image is preprocessed and the different types of food are divided from each other through segmentation techniques, *3*) robust and discriminative features are then extracted, *4*) classification of food items takes place, and *5*) the volume of each food item is calculated and the energy and nutrients of the depicted meal are estimated using appropriate nutritional databases. Each phase has been implemented in a variety of ways to optimize the meal's calories and nutrient estimation results. A comparison of the different methodologies tried in each phase is a demanding task, since very different approaches have been adopted and several evaluation metrics exist (13). Moreover, image datasets used in such systems also vary significantly; thus, comparison remains an open challenge. Although automatic record keeping based on an IBFRS also has limitations, such as measurement error because of real-life conditions in the photograph setting (low lighting or other irrelevant objects on the scene) or because the user must remember to take a picture of the meal and its leftovers, research efforts aim already at minimizing the sources of such errors—for example, with the use of automatic text messages on the user's device (14). Thus, IBFRS can be an easy to use and objective tool for dietary assessment.

To compare different approaches on food image recognition, datasets on which researchers can test their methodologies have been recently released publicly. An extensive overview of these datasets along with a critical discussion of related advantages and limitations will be described below. Moreover, information about the implementation of the several phases of dietary assessment systems for each of the reviewed papers will be presented focusing on their critical comparison. Open issues that should be tackled in the near future to further optimize the performance and adoption of IBFRS in dietetic professional practice will be thoroughly examined and discussed.

The aim of this review is to present IBFRS combining a camera of the user's mobile device with computer vision methods for supporting dietary assessment. We investigated whether IBFRS can be more objective, user-friendly, and more educational than manual record or recall methods. All statistical and computational aspects of the topic are presented in a comprehensive manner so as to encourage dietitians to adopt such systems. Towards this goal, we also present current applications of IBFRS in different areas of dietetic and nutrition practice. Finally, we highlight that improvement in IBFRS will take place through interdisciplinary efforts at the intersection of computer science with nutrition and dietetics, so that the challenges of such systems are gradually set aside.

## Methods

### Search strategy

In July 2021, PubMed (for the time period between 1 July 2016 until 1 July 2021) and Scopus (for the years 2016–2021) were searched by combining Boolean operators with suitable keywords. In particular, the following query was formed: (food AND image AND (classification OR recognition OR segmentation OR (dietary AND assessment))). A study was eligible when *1*) it was peer reviewed, *2*) it was written in English, and *3*) it included the performance of either the segmentation or the classification or the volume estimation phase of a dietary assessment system based on food images.

Two authors (KVD and KP) reviewed the articles and decided on their inclusion or exclusion. A study was excluded when the title implied that there was no association with the image-based food-recognition task and the study was out of the scope of this review. Studies that included IBFRS without presenting their performance in at least 1 of the segmentation, classification or volume, calories, and nutrients estimation phase were also excluded. In image classification, for example, 1 common performance metric is accuracy, which is the number of correctly classified images divided by the total number of examined images of a dataset. Studies that were published before 2016 were excluded in this systematic review, because, since then, image-processing tasks with a large volume of input data have been solved in short runtimes, which can also be used for mobile applications, with the adoption of graphics processing units (GPUs) that can accelerate complex, parallel calculations in a large volume of data (15).

**FIGURE 1:**
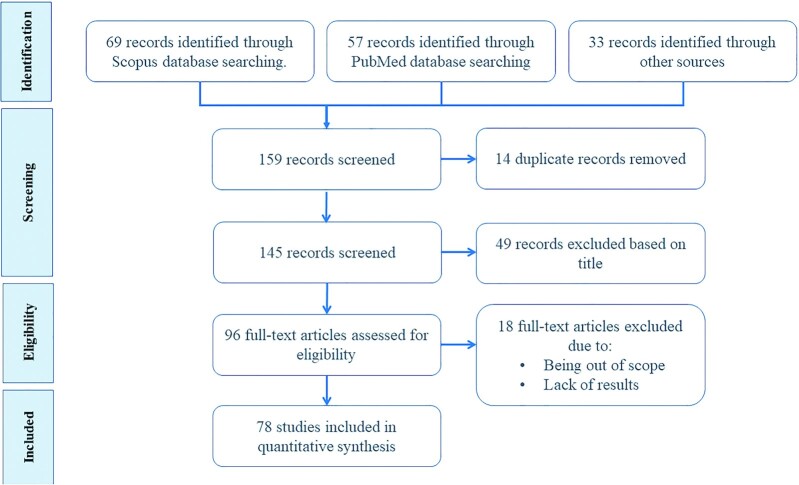
Search strategy for publications included in this systematic review for image-based food-recognition systems. In this review, 159 titles were identified when the query (food AND image AND (classification OR recognition OR segmentation OR (dietary AND assessment))) was formed in the PubMed and Scopus databases or from other sources that the reviewers had identified in the past. After removing 14 duplicates, 145 studies were screened by title and 49 were removed, since they were irrelevant to the subject of this study. Thus, 96 studies were assessed for eligibility by full-text reading. Eighteen studies were excluded because they were out of scope or they did not present the performance of the image-based food recognition system presented. Finally, 78 studies were included for full-text review.

In this review, 159 titles were identified when the above-mentioned query was formed in the PubMed and Scopus databases and from other articles that the reviewers had already studied in the past. After removing 14 duplicates, 145 studies were screened by title and 49 were removed, since they were irrelevant to the subject of this study. Thus, 96 studies were assessed for eligibility by full-text reading. Eighteen studies were excluded because they were out of the scope of this review or they did not present the performance of the IBFRS presented. Finally, 78 studies were included for full-text review that included IBFRS for assessing dietary intake. The search strategy followed is outlined in **Figure 1**.

## Results

### Architecture of image-based food-recognition systems for dietary assessment

**FIGURE 2:**
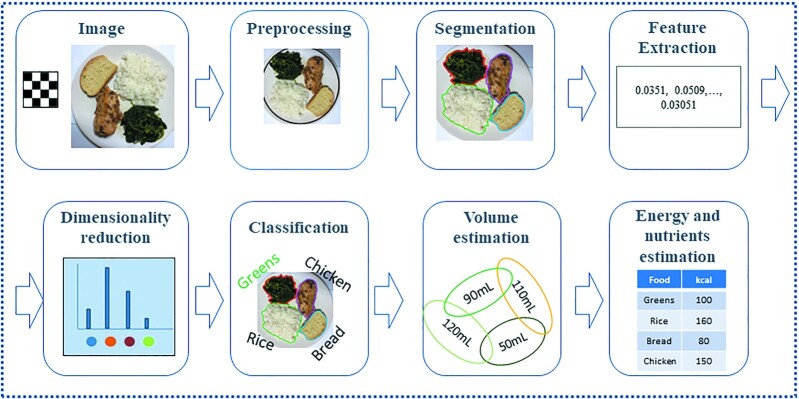
Architecture of image-based food-recognition systems for dietary assessment. The flow of the dietary assessment systems combining a camera of the user's mobile device with computer vision methods is as follows: *1*) the user takes a photograph of the upcoming meal with a camera of his/her mobile device, *2*) the image is preprocessed, *3*) the different types of food are divided from each other through segmentation techniques, *4*) robust and discriminative features are then extracted, *5*) the most important features are selected and are given as input to the next phase of the system, *6*) classification of food items in food categories takes place, *7*) the volume of each food item is calculated, and *8*) the calories and nutrients of the depicted meal are estimated using appropriate nutritional databases.

The architecture of such dietary-monitoring systems based on the camera of a mobile device is shown in **Figure 2**. The flow of such systems is as follows: The user first takes a photograph of the upcoming meal with a mobile camera, such as the camera of his mobile phone (16) or his smart watch (10) or his smart button (17). Then, the image is preprocessed and the user may insert additional information by drawing polygons (6). The different types of food or drinks (18) are then divided from each other in separate regions as segmentation takes place. The extraction of robust and discriminative features and classification follow the phase of segmentation. In the case of extraction of hand-crafted features, a series of features are extracted from each segmented area and are fed to a traditional machine-learning classifier, which decides what kind of food is represented by each food region (19). In the case of using convolutional neural networks (CNNs) for feature extraction (6), the intermediate or last layers of the CNNs extract the appropriate features. These CNN-extracted features are then fed either to shallow machine-learning classifiers, such as support vector machines (SVMs) or to the last layers of the CNNs for classification (6). Sometimes, between the feature extraction and classification phases, the dimensionality reduction phase takes place (20), which can improve the accuracy of the classification task by reducing the number of input features. Finally, the volume of each food region (21) is calculated and the energy or nutrients of the depicted meal are estimated using available nutritional databases (22).

### Publicly available food datasets

To compare different IBFRS on dietary assessment, publicly available food datasets (PAFDs) have been released recently, which are used as input to these systems. The main attributes of the PAFDs for food image recognition are presented in **Table 1**.

**TABLE 1:** Publicly available food datasets used as input to image-based food recognition systems

No.	Name	Year	Food categories, *n*	Total number of images, *n*	Food items in each image	Cuisine	Reference
1	Pittsburgh Fast-food Image Dataset (PFID)	2009	61	1089	Single	Fast food	(23)
2	UEC-Food 100	2012	100	10,000	Single and multiple	Japanese	(24)
3	NTU-FOOD	2012	50	5000	Single	Multiethnic	(28)
4	UNICT-FD889	2014	889	3583	Single	Multiethnic	(26)
5	Food-101	2014	101	101,000	Single	Multiethnic	(29)
6	UEC-Food 256	2014	256	31,397	Single and multiple	Multiethnic	(25)
7	Ambient Kitchen	2014	12	1800	Single and multiple	Multiethnic	(30)
8	UPMC Food-101	2015	101	90,840	Single	Multiethnic	(31)
9	Dishes	2015	3832	117,504	Single	Multiethnic	(32)
10	Menu-Match	2015	41	646	Single and multiple	Asian, Italian	(33)
11	FooDD	2015	23	3000	Single and multiple	Multiethnic	(34)
12	UNIMIB 2015	2015	15	2000	Multiple	Italian	(35)
13	Instagram800K	2016	43	808,964	Single and multiple	Multiethnic	(36)
14	UNICT-FD1200	2016	1200	4754	Single and multiple	Multiethnic	(27)
15	UNIMIB 2016	2016	73	1027	Multiple	Italian	(6)
16	EgocentricFood	2016	9	5038	Multiple	Multiethnic	(17)
17	VIREO Food-172	2016	172	110,241	Single and multiple	Chinese	(37)
18	FOOD-5K	2016	2	5000	Multiple	Multiethnic	(38)
19	FOOD-11	2016	11	16,643	Multiple	Multiethnic	(38)
20	NTUA-Food 2017	2017	82	3248	Single	Multiethnic	(39)
21	ECUST Food Dataset	2017	19	2978	Single and multiple	Multiethnic	(40)
22	Madima 2017	2017	21	21,807	Multiple	Central European	(43)
23	Food524DB	2017	524	247,636	Single and multiple	Multiethnic	(41)
24	ChineseFoodNet	2017	208	192,000	Single and multiple	Chinese	(44)
25	Eating Occasion Image to Food Energy	2020	21	96	Multiple	Multiethnic	(45)
26	ChinaFood-100	2021	100	10,074	Single	Chinese	(22)
27	VIPER-FoodNet	2021	82	14,991	Multiple	Multiethnic	(46)

The first PAFD is the Pittsburgh Fast-food Image Dataset (PFID), which was released in 2009. The PFID depicts 101 different foods from popular fast-food chains. Since many food items can be confused with others as they may differ only in terms of the filling, the 101 food categories have been merged into 7 broad fast-food categories (23).

The UEC-Food 100 dataset depicts 100 different Japanese food categories (24), whereas the UEC-Food 256 dataset, which is an extension of the UEC-Food 100 dataset, depicts 256 different food categories from Japan and other countries (25). Both datasets contain single and multiple items per image. The existence of multiple items in the image encumbers the food-recognition task.

The UNICT-FD889 dataset consists of 889 food categories of different national cuisines (e.g., Italian, English, Thai, Indian, Japanese, etc.) (26), whereas UNICT-FD1200, which is an extension of UNICT-FD889, consists of 1200 distinct dishes. Each food category has been acquired under varying geometric and photometric conditions (27).

The NTU-FOOD dataset depicts 50 categories of multiethnic food. Each category contains 100 images, either from the user's mobile phone or from Internet web collections (28).

The ETHZ Food-101 or Food-101 dataset depicts the 101 most popular food categories from the foodspotting.com site. The Food-101 dataset is a very large food-image dataset with 101,000 images. The training set of images has not been cleaned; therefore, they still contain some amount of noise, either as intense colors or as wrong labels (29).

The Ambient Kitchen dataset contains 1800 images of 12 food ingredients that have been used to cook a full meal (30).

The UPMC Food-101 dataset (31) depicts the same 101 categories as the Food-101 dataset (29), but they are chosen from the results of the Google Search Engine when using the name of the class followed by the word “recipe”. The images of the UPMC Food-101 dataset are less noisy and more relevant to the depicted class than the Food-101 dataset (31).

The uniqueness of the Dishes dataset is that it includes restaurant and geographic information about the depicted food dishes. The images of 3832 categories of restaurant dishes have been collected from a restaurant reviewing site (32).

The Menu-Match dataset consists of 41 categories of food from 1 Asian, 1 Italian, and 1 soup restaurant. The dataset contains 646 images of 1386 food items divided in 41 food categories. The uniqueness of the Menu-Match dataset lies in that it contains additional nutritional information and images depicted in a real setting (33).

The FooDD dataset consists of images of 23 food categories taken with different cameras under different lighting conditions. The food images have been divided into 2 different collections, single and mixed food portions (34).

The UNIMIB 2015 database is composed of 2000 tray images with multiple foods from a canteen environment and contains 15 food categories. UNIMIB 2015 is the only PAFD that can be used for food recognition and leftover estimation. UNIMIB 2015 contains images from the beginning as well as the end of the meal (35). UNIMIB 2016 (6) is created with the same principles as UNIMIB 2015 and it is composed of 1027 tray images with multiple foods that are divided in 73 food categories.

The Instagram800K dataset is collected using the Instagram API. A total of 808,964 images with the most popular 43 food-related tags, such as #lunch and #foodie, and the most popular 53 food items, such as #pasta and #steak, and their metadata are included in this dataset (36).

EgocentricFood contains images taken by a wearable vision camera, including a total of 9 different food-related classes totaling 5038 images and 8573 bounding boxes indicating the location of the food item in the photo. This dataset is the first PAFD that can be used for 2 different tasks: food recognition and food detection, as well as localization inside the plate (17).

VIREO Food-172 contains 172 categories of Chinese food and 353 labeled ingredients. The singularity of this dataset is that it includes both food category and ingredients (37).

The FOOD-5K has been created for the task of classifying food from nonfood images (38).

FOOD-11 contains images of 11 categories, such as dairy, bread, egg, dessert, meat, fried food, pasta, seafood, rice, vegetables/fruit, and soup (38).

NTUA-Food 2017 (39) contains images that are collected from the web, the Food-101 dataset (29), and the UEC-Food 256 dataset (25). The images are organized in 8 broad categories with macronutrient content that leads to different postprandial fluctuations of blood sugar, as determined by the Hellenic Diabetes Society (39). The NTUA-Food 2017 dataset in the only PAFD that addresses the dietary needs of people with diabetes mellitus.

The ECUST Food dataset contains food images divided in 19 categories taken with the camera of a mobile phone from 2 different views, top and side. The images also contain a coin as a fiducial marker, which can be used for volume estimation (40).

The Food524DB dataset (41) consists of 247,636 images divided in 524 categories taken from the existing datasets VIREO Food-172, Food-101, Food50 (42), and UEC-Food 256.

The Madima 2017 dataset consists of 21 categories of food depicted in images of 80 meals. This dataset provides segmentation and recognition maps, as well as information that can be used as ground truth for volume estimation (43).

The ChineseFoodNet dataset is thus far the largest image dataset for Chinese food classification, consisting of 192,000 images divided in 208 categories (44).

The Eating Occasion Image to Food Energy dataset contains 96 images, which contain 834 single items with the associated calories in each food item (45).

The ChinaFood-100 dataset is created for classification purposes of Chinese food (22). This dataset contains rich information about each food category including its calories, proteins, fat, carbohydrates, vitamins, and micronutrients.

The VIPER-FoodNet dataset consists of 14,991 images of multiethnic food categorized in 82 classes (46).

### Image-based food-recognition systems

In the following subsections, information about the implementation of the several phases of IBFRS is presented.

### Image depiction

As described in the Introduction, initially, the user depicts the meal to be consumed with the camera of his/her mobile device. The camera of the mobile device can affect the results of the food-recognition task in terms of its lens, hardware, and software (34). One (16, 47, 48) or 2 (21, 49) photographs taken from different angles can be used for the depiction of the meal. Using only 1 image can reduce the user's burden but does not carry sufficient information and makes the 3D reconstruction and volume estimation phases more difficult. When 2 images are taken, the first image, taken from the top, can be used to estimate the food area, and the second image, taken from the side, can be used to estimate the height. However, when capturing the dish from the side, occlusions may occur, while the assumption that all the food items have a constant height may introduce large errors in volume estimation (50). Therefore, another approach was introduced that takes 2 images from the sides of the plate and then uses a reference object to match key points between the 2 images. The disparity among the pixels of the 2 images has been used to provide the depth of the food items (50).

Depending on the food recognition system, to enable food volume estimation the user might have to include in the photo a reference object of known dimensions, such as a fiducial marker (51), a coin (52), a credit card (50), the user's thumb, a circular plate, bowl, or cup. A fiducial marker, a coin, or the user's thumb may be used for spatial calibration of the camera. To deal with varying lighting conditions, a colorful fiducial marker may be placed in the image for photometric calibration. A circular plate, bowl, or cup of known dimensions may be used to enable volume estimation by multiplying the width of the food item with the known depth of the plate, bowl, or cup. The use of a credit card, although adopted in some studies, should be avoided for security reasons. To optimize the food-segmentation process the user might be asked to draw bounding boxes, polygons (6), or mark specific touch points in the scene.

To simplify food-volume estimation, some applications also use a thermal camera for the representation of the meal, since it can provide information about the depth of the food items (49, 53). This approach, although it requires additional equipment, might prove to be very accurate in terms of volume error. Other types of cameras, such as laser, multispectral, and hyperspectral cameras, have been successfully adopted for food items and ingredients recognition (54–56). For example, oil and vinegar dressings in salads have been recognized with a multispectral camera, achieving high values of accuracy (56).

To achieve better recognition performance many existing approaches leverage additional contextual information, such as geographical information (GPS coordinates from the user's mobile phone) about the restaurant in which the meal was depicted (47) or temporal information about the eating time (57) or information about food sharing when several persons are involved (58).

### Segmentation

The accurate estimate of caloric content in the user's meal is primarily dependent on well-defined food regions. The first step is to extract dish regions. Next, food regions that are expected inside the detected dish region are localized.

The challenges for segmentation inherent to food images are numerous. Often, different ingredients are mixed and cannot be optically divided, such as ingredients in a salad, soup, or burger, or ingredients mixed with rice or pasta. Small variability in the color, shape, and texture of different food items can also impede segmentation, such as small differences between toasted bread and roast beef.

Another challenge for segmentation in general is the lack of widely accepted evaluation metrics. One standard way of evaluating the accuracy of an image segmentation algorithm is to manually segment the image of interest and compare it with the algorithm-generated segmented regions in terms of overlap (59). However, manual segmentation is particularly subjective and very unlikely to be reproducible. Moreover, an accurate evaluation of segmentation methods should include sensitivity and specificity metrics, such as the Dice Similarity Coefficient (60), and not solely absolute volume-based statistical evaluations, which is unfortunately not the case for most of the published articles.

### Dish detection

For the detection of the dish, edge detection methods can be used, which are easy to implement but susceptible to noise and artifacts, as well as sensitive to the orientation of the boundary (48). Ellipse-based and circle-based Hough transform has also been used for dish detection, since the shape of the dish does not vary significantly (35).

### Segmentation techniques for food items

**FIGURE 3:**
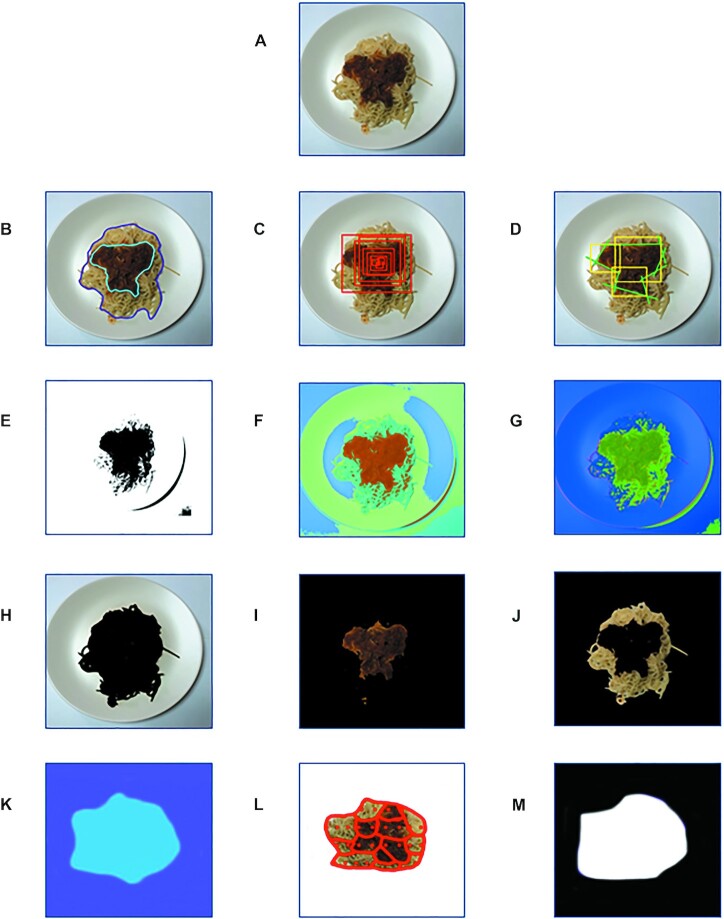
Summary of segmentation methods used for the food-segmentation task in image-based food-recognition systems. (A) Initial image of plate with user's meal. (B) Manual segmentation of initial image. The user draws a line/border/polygon manually around each food item. (C) Hierarchical segmentation of initial image. Hierarchical segmentation starts with an initial over-segmentation, where almost every pixel defines a different region, and gradually constructs finer segmented regions based on a specific criterion. (D) Saliency-aware segmentation of initial image. Saliency-aware segmentation uses spatial, color, and statistical features of food areas to enhance food regions and suppress nonfood regions. (E) Thresholding segmentation of initial image. A binary image is created where all pixels with color intensity above the predefined threshold are depicted with 1 color (e.g., white) and could indicate the background area, and all pixels below the threshold are depicted with another color and could indicate the food area. (F) Clustering segmentation of initial image. Pixels of food items are grouped into groups/clusters depicted by different color (e.g., 4 groups/colors are created in this example). (G) Segmentation of initial image based on Sobel operator. Edges of food items can be estimated by applying the Sobel operator to every pixel of the image (i.e., convolving the matrix on the left with the respective 3 × 3 matrix of the image for every pixel). After the convolution, areas in the image where the color intensity of the pixels change rapidly denote the border of a food item. (H) Color/texture-based segmentation of initial image. Color/texture-based segmentation assumes that regions of pixels that share similar color/texture properties in the image correspond to meaningful objects. The first cluster depicts the plate and the background. (I) Color/texture-based segmentation of initial image. The second cluster depicts the sauce. (J) Color/texture-based segmentation of initial image. The third cluster depicts the spaghetti. (K) Thermal clustering of initial image. Dynamic thermal thresholding can be applied for the discrimination of food from the plate and the background, since food is hotter than the other elements of the image. (L) Region-based segmentation of initial image. Starting points (seeds) of different areas are depicted with dots. Then, the algorithm expands the initial areas around the starting seeds with pixels in their neighborhood that fulfill a criterion based on a homogeneity metric. (M) Segmentation based on CNNs on initial image. CNNs were used for food localization by identifying the pixels that might belong to a food item. A binary image is created where all pixels that are categorized by the CNN as a food item are depicted with white color, whereas all the remaining pixels that are categorized as background are depicted with black color. CNN, convolutional neural network.

Several segmentation methods have been implemented for food recognition (**Figure 3**), which can be divided in the following main categories: manual (6), thresholding (61), clustering (48), region-based (53), graph-based (62), based on a Sobel operator (63), hierarchical (49), color-based (52), texture-based, thermal thresholding (53), and based on CNNs (17).

In manual segmentation with polygons (6), the user draws boundaries around the food items with polygons. Manual segmentation is easily implemented, but, on the other hand, it is dependent on the user and time-consuming. Segmentation using the intervention of the user in the form of touch points has also been proposed (50). The results show that this minimal user intervention can improve the precision of segmentation.

Thresholding methods assign pixels to different levels of color intensity (61). These color levels may be defined by an expert or derived by an analytic expression. These methods are simple to implement; however, in the case of noisy images, they produce unsatisfactory segmentation results. Moreover, the optimal threshold is difficult to calculate since it is dependent on several factors, such as the image properties and the type of camera.

Learning-based segmentation techniques gather pixels with alike properties, such as intensity and location, into sets that represent distinct food items. Such segmentation methods are based on k-means clustering (64) and divide all of the pixels within the target dish bounding box into foods, dish, and background, assuming that the order of the regions from the center to the outside are always foods, dish, and background (48). Clustering methods can be very effective, especially when the shapes of the food items are nonconvex and the background is heterogeneous.

Region-based segmentation methods can be further divided into 2 subgroups: region growing and graph-based methods. Region growing methods start with the selection of a starting point (seed) location by the user, then combining the mean and SD of the color intensities of the pixels within an area, with a homogeneity metric, a decision is made whether a new pixel is going to be included into the area depending on its similarity with the statistics of the aforementioned area. Nonparametric region growing/merging segmentation has been used for food recognition, although region growing methods suffer from the fact that their results may differ significantly with a different homogeneity criterion or a different initial seed location (65).

Graph-based segmentation methods, such as normalized cuts (62), super pixel segmentation (66), and graph-cut segmentation (67), are implemented by creating a weighted graph (i.e., a structure consisting of a set of objects connected with edges in pairs, where each vertex corresponds to a pixel or a region of the image and the edges represent the strength of the similarity between the vertexes). After the graph construction, edges are eliminated in order to minimize an energy function. Graph-based segmentation methods produce, in general, optimal results but fail to address noisy images and are not very robust.

Boundary-based methods, such as active contours, start initially with a curve around an object and move towards the object's edges according to the value of an energy function. A major drawback of active contours methods is that they rely heavily on the location of the initial curve (68).

Segmentation based on the Sobel operator has also been proposed for food segmentation (63). Edges of food items can be estimated by applying the Sobel operator to the whole image. Areas in the image where the color intensity of the pixels change rapidly denote an edge (i.e., a border of a food item). The computation of the Sobel operator is based either on a gradient vector or its norm and is inexpensive, but the calculation of the gradient approximation is relatively coarse, especially for high-frequency variations in the image.

Hierarchical segmentation has also been used in several diet-monitoring systems (49, 53). Hierarchical segmentation starts with an initial over-segmentation, where almost every pixel defines a different region, and gradually constructs larger segmented regions that depict food items. The effectiveness of hierarchical segmentation highly depends on the selection of parameters, such as the scale, the measure of goodness-of-fit, and of segmentation complexity.

Color-based segmentation assumes that regions of pixels that share similar color properties in the image correspond to meaningful objects (50, 52). A drawback of these methods is that segmentation results differ when different color spaces are used, and that food items with similar color with the plate or the background may not be distinguished from them.

Texture-based segmentation methods use the features of the microstructure of the food item. Texture features, such as Haralick's co-occurrence matrices, might be computationally intensive, and texture-based segmentation might be coarse and may need refining. Therefore, texture-based segmentation methods are often part of several-phase segmentation techniques (34).

In the case that foods and their containers have similar colors and textures, defining food boundaries might be a very complex task. To tackle these problems, saliency-aware segmentation has been proposed, where spatial, color, and statistical features of food areas are used to enhance food regions and suppress nonfood regions. A limitation of this method is the inability to locate food items when no container is present in the image (69).

When a thermal camera is available, dynamic thermal thresholding can be applied for the discrimination of food from the plate and the background (49, 53), since food is supposed to be hotter than both of these. The combination of color and thermal images to segment food items can ameliorate the segmentation results, especially in cases where the plate color or shape is similar to that of a food item. However, the addition of a thermal camera to a smart phone might be inconvenient for the user.

Another recently proposed approach in segmentation is based on CNNs. CNNs can leverage image segmentation by learning complex spatial patterns. The existence of large annotated datasets has led to the great improvement in the performance of these models. CNNs have been used for food localization by identifying the pixels that might belong to a food item (17). A specific layer of the CNN, a Global Average Pooling layer, is used to create food activation maps [FAMs; i.e., heat maps of probabilities that denote if a pixel belongs to a food item (foodness scores)]. The performance of these methods seems to be superior to traditional segmentation methods (17).

### Feature extraction

The challenges associated with classifying correctly different food classes lie also on the features/descriptors that describe the respective food regions. A feature/descriptor is a characteristic value that describes a certain visual property of an image. A feature is global if it characterizes the whole image or local if it characterizes specific regions of the image. The extracted features from the regions of interest should be similar for items of the same food class under different lighting or serving conditions and different for items of different food categories. Several kinds of features/descriptors have been implemented for the food-recognition task trying to adopt the above principles and are divided into 2 main approaches—hand-crafted descriptors or descriptors extracted from the inner layers of CNNs.

Hand-crafted features can be further divided into color, texture, size, and shape features. Color features are numbers that denote the color intensity of a pixel. For example, the triple (0.8, 0.3, 0.3) in red-green-blue (RGB) color space denotes a shade of red, since the first component of the triple, which refers to the red channel, has a large value near 1. The values of the 3-color components in RGB lie in the range [0, 1]. Color features are rotation invariant features. Most commonly used color spaces in food recognition are RGB, hue-saturation-value (HSV), and LAB (L for lightness and A and B for the color opponents green-red and blue-yellow) (70–72). While RGB is the most commonly used color space, HSV is particularly intuitive and LAB is device independent.

Another effective representation of the color content of an image is color histograms—namely, RGB, HSV, and LAB histograms (73). A color histogram is a diagram that shows the number of pixels that have a specific color value for each of the available color values. Color histograms are easy to compute and intuitive, but have high dimension, do not take pixel spatial information into account, and are sensitive to noise.

Other representations are color moments, which encode both shape and color information and are effective under changing scaling, rotation, and lighting conditions. Color moments are numbers that measure the distribution of color in an image, such as the mean, SD, and skewness of the color intensities. However, color moments cannot handle occlusion very successfully and do not take into account spatial information. For food-recognition purposes, color moments have been also combined with Local Orientation Descriptors (52).

Computer vision systems inspired by the human visual perception system also use texture features to identify objects. Image texture may be described by the calculation of a set of statistical measures from the distributions of pixels’ color intensities values, taking into account spatial relations between pixels. First-order statistics are properties of individual pixel values, such as average and variance, whereas second- and higher-order statistics are properties of 2 or more pixel values, taking into account the spatial interaction between pixels, such as co-occurrence features and gray-level differences. Other common texture features are local binary patterns (LBPs) (19, 70, 71) and their variation, Pairwise Rotation Invariant Co-occurrence LBP (PRICoLBP) (27). LBPs can be calculated by taking the histogram of the binary words that are produced by comparing each pixel's intensity with the intensity of its neighbors. PRICoLBP's advantage over LBP is that it is invariant to global illumination changes. Texture primitives, such as edges, may also be extracted to describe an object with appropriate filters such as a Laplacian-of-Gaussian or a Difference-of-Gaussian filter. Texture features described above are meaningful and can be extracted from any region of any shape without losing significant information. On the other hand, they are sensitive to noise and distortions. Spectral-based texture features, such as Binary Gabor Patterns and the MRS4 filter bank (70), capture the frequency content of the image. Spectral-based features are robust but have no semantic meaning. Another category of features, Textons and anti-Textons, have been also used for food recognition (27). Textons are hypothetical elements of pre-attentive perception, such as line segments and elongated blobs, that can yield texture discrimination. Anti-Textons encode spaces between Textons. Textons and anti-Textons are invariant to positional and scaling transformations. Other features, such as local phase quantization (LPQ), which are robust to image blurring, and local configuration pattern (LCP), which is a rotation invariant descriptor, have been combined and it has been proven that they are highly discriminative for food recognition (70). Other texture features, such as histogram of oriented gradients (HOG), and its variation RootHOG, and Gradient Orientation Spatial-Dependence Matrix (GOSDM) descriptors explore local structures with low-level characterization, such as coarseness and contrast, and basic visual elements, such as dots, lines, and circles (25, 74).

Shape features, such as physical area, diameter, and eccentricity, are less frequently used, since they presume that food items have regular shapes.

Deep learning architectures, such as CNNs, have seen a rapid increase in their use recently due to the availability of large image datasets and fast computing hardware, such as GPUs. CNNs are multi-layered artificial neural networks inspired by the way the visual cortex works and are used for feature extraction and supervised classification. They were established in ImageNet Large-Scale Visual Recognition Challenge (ILSVRC) 2012, where they improved classification accuracy compared with hand-crafted feature extraction methods (75). To avoid training CNNs, which are computationally very expensive and require large datasets, a pretrained CNN model can be used for feature extraction and then another simpler classifier can be used for classification (transfer learning) (70, 76). In the current literature, the CNN-based features are obtained as the output of the intermediate (6) or output (76, 77) layers of trained deep CNNs and make significant improvements over the traditional descriptors (6, 76, 77).

The CNNs’ 3 main types of layers are convolutional, pooling, and fully connected. Convolutional layers consist of different kinds of filters that represent certain features, such as edge orientation and frequency. The deeper the convolutional layer, the more complex are the learned features. The pooling layers decrease the dimensions of the input data. The fully connected layers compose higher-level representations of their input data from the other layers (75).

The most commonly used CNNs for feature extraction are the 3 winners of the ILSVRC: AlexNet (2012), GoogLeNet (2014), and ResNet (2015). AlexNet consists of 5 convolutional layers and 3 fully connected layers. It provides less complex features, since it has only 5 convolutional layers, but is faster in terms of running times (78). GoogLeNet has 22 layers, whereas ResNet is the deepest, since it has 152 layers. GoogLeNet, although deeper than AlexNet, has less parameters (4 million) than AlexNet (60 million) (79). The runner-up at the ILSVRC 2014 was the VGGNet, a CNN that consists of 19 layers. VGGNet is preferably used for feature extraction, since it has 16 convolutional layers and can obtain very complex representations of an image, but is very slow in terms of training times, since it has 138 million parameters (80). ResNet, although deeper than VGGNet, is less complex than VGGNet, since it is based on a “skip connections” technique (81).

### Dimensionality reduction

Classification results of food images in dietary intake monitoring systems can be improved when the dimension of the extracted feature vector is reduced. Moreover, such a limited amount of descriptors could be ideal for applications on mobile devices, which have limited computational power. The use of the Bag-Of-Features (BOF) model (20, 27, 52), which is inspired by the Bag-Of-Words model for text classification, has been reported to greatly improve classification accuracy in food-recognition tasks. The BOF model groups together the extracted visual features in clusters. The centroids of these clusters are called codewords. The frequency of the codewords in an examined image are used to represent this image in the BOF model.

Dimensionality reduction is also implemented using the Fisher Vector approach, or its descendants, such as the Vector of Locally Aggregated Descriptors (VLAD), since it is an extension of the BOF model, which can achieve even better results in classification, due to the fact that it uses an alternative patch encoding strategy based on the deviation of patches from a universal Gaussian mixture model. Another advantage of the Fisher Vector representation is that it achieves very good results even with linear classifiers and can be compressed with a minimal loss of representation accuracy (25, 82).

Another method that has been implemented is the Orthogonal Matching Pursuit (OMP), which can be used for the construction of overcomplete dictionaries for sparse representations. The update of the dictionary is performed in parallel with the update of the sparse representation; thus, the convergence is accelerated and the flexibility of the method is increased (73).

### Classification

Classification of food images depends heavily on the descriptors used. Moreover, the values of the classifiers’ hyperparameters influence significantly the final result. The food image datasets used for the training of the algorithms also play an important role in the final classification result. To achieve good classification performance, the designer of an IBFRS should take all these aspects into account.

Several “shallow” classifiers, such as artificial neural networks (ANNs), SVMs, k-nearest neighbors (KNN), naive Bayes, and random forests (RFs), have been used in combination with hand-crafted features or CNN-based features for classification of food images (**Table 2**).

**TABLE 2:** Summary of classification methods used for the food-recognition task in image-based food-recognition systems

Classification method	Depiction	Pros	Cons
Artificial neural network (ANN)	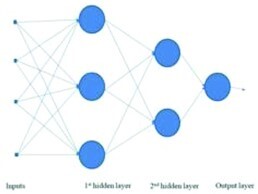	Can achieve high recognition accuracy over 80% even when there is a nonlinear relation between the input and the output	Dependent on many parameters Low speed of computation Lack of interpretation of results
Support Vector Machine (SVM)	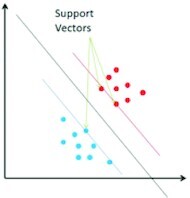	Can achieve high recognition accuracy over 80% even when there is a nonlinear relation between the input and the output	Binary classifiers
Naive Bayes (NB)	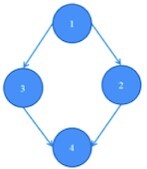	Can take into account prior knowledge about the domain in interest	Less accurate than other machine-learning algorithms, such as ANNs Unsuitable for large number of features
K-nearest neighbor (KNN)	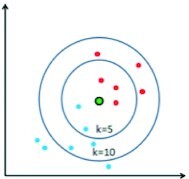	Easily implemented without the need for large computational resources during execution	Sensitive to the choice of parameters
Random forest (RF)	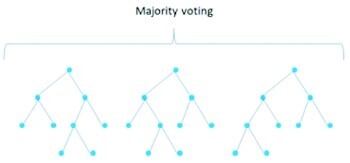	Accuracy Speed Comprehensible by humans	—
Convolutional neural network (CNN)	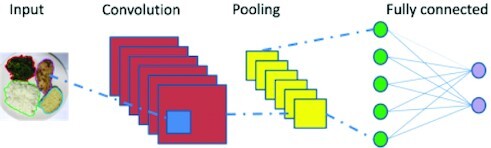	Optimal results	Large datasets for training

ANNs are inspired by the way biological neural networks work and are typically organized in weighted interconnected layers (71, 83). ANNs may perform well, even when there is a nonlinear relation between the input and the output. However, neural networks’ performance depends upon several parameters, such as the input features, the activation functions of the neurons, the weights of the connections, and the overall network architecture. Moreover, they are characterized by a low speed of computation and the lack of ability to explain the output so that the human expert can understand the inner laws of the results.

SVMs (20, 52, 66, 76, 84) and their variations (19, 70, 85) represent the input instances in such a way that the instances of different classes are separated by a clear gap that is as wide as possible in the transformed feature space. SVMs can also represent a nonlinear relation between the input and the output; they are, however, binary classifiers. Therefore, for the food-recognition task, which is a multiclass problem, the need to transform the problem to a set of multiple binary classification problems emerges.

Statistical approaches, such as the Bayesian networks (71), are characterized by the ability to take into account prior knowledge about the domain of interest, in terms of structural relations among its features. Bayesian models are often less accurate than other more sophisticated artificial intelligence methods, such as ANNs and SVMs. Moreover, Bayesian models are not suitable for datasets with many features, since the construction of a very large network is impossible in terms of computation time and memory space.

The KNN classifier (27) assigns an object to the class that is the most common among its k nearest neighbors, where k is a small integer. KNN classifiers are sensitive to the choice of the integer k and the distance function that defines the k nearest neighbors.

RFs are ensembles of decision trees (type of tree C4.5). An ensemble is a group of classifiers that are combined with a rule, such as majority voting, to assess an instance of the examined dataset. RFs achieve a very good combination of accuracy and speed and are comprehensible by humans, but, since most decision tree algorithms divide the output space in hyperrectangles, their performance is deteriorated for problems that require diagonal partitioning (86).

On the other hand, deep learning algorithms, such as CNNs, can be used for the food-classification task (6, 17, 22, 47, 48, 87–91). Pretrained CNNs on a large image dataset can be retrained (fine-tuning) in order to differentiate several layers, so that they are adjusted to the specific food-recognition task. Recently, even ensembles of CNNs have been constructed for classification (92, 93). CNNs have shown superior results over other shallow classifiers in many PAFDs, as will be shown in the following section "Image-based food-recognition systems using publicly available datasets as input".

### Volume, calories, and nutrients estimation

Volume, calories, and nutrients estimation is the less addressed phase of an IBFRS. Challenges that hinder achieving excellent performance in this step are the lack of appropriate annotated datasets and the difficulty in obtaining depth information from a 2-dimensional image.

A simple approach for the stage of the volume estimation is to multiply the height with the depth of the food object (94). When a depth camera is available, the differences of depth values between the empty tray and the tray before/after eating can be used to estimate the volumes of the foods (95). When a reference object is included in the photo of the meal, such as the thumb of the user or a coin or a reference card, comparing the reference object dimensions with meal dimensions can lead to volume estimation (48, 52). Another approach is to match each food object to a predefined shape, such as a cylinder or a prism (16). A structured light system consisting of a laser module and a diffraction lens was also implemented for volume estimation (55).

After volume estimation, the total calories of the depicted food items can be estimated using the volume of each item multiplied by its density and the calories that match a predefined mass in appropriate nutritional databases (37, 96–100). In another approach, calorie estimation was conducted using a Support Vector Regressor with a BOF model on hand-crafted features (color and texture) (33). Nutrients, such as protein, fiber, calcium, vitamin C, and iron, were estimated by using as input the top-5 food categories that the classifier recognized in the previous step of the IBFRS and appropriate food databases with information for each food category (22). Since excess calorie and salt intake may lead to cardiovascular disease, calorie content along with sodium content were estimated from food images with a multi-task CNN. The multi-task CNN was trained using an appropriately annotated dataset with ingredients and salinity information for each image (101).

### Performance evaluation metrics

The performance of IBFRS for dietary assessment is evaluated using several metrics, such as the Accuracy (Acc), the Precision (Pr), the Recall (Re), the Mean Average Precision (MAP), the F-measure, and the Mean Absolute Percentage Error (MAPE) described in **Table 3** (13). In image classification, accuracy is the number of correctly classified images divided by the total number of examined images. In binary classification, where there are 2 classes, the positive and the negative class, to better understand the performance of the classifier 4 numbers are important: true positives (TPs), true negatives (TNs), false positives (FPs), and false negatives (FNs). TP denotes the number of instances that have a positive label and are classified as belonging to the positive class, TN denotes the number of instances that have a negative label and are classified as belonging to the negative class, FP denotes the number of instances that have a negative label and are classified as belonging to the positive class, and FN denotes the number of instances that have a positive label and are classified as belonging to the negative class. In binary classification, accuracy is the sum of TP and TN divided by the sum of TP, TN, FP, and FN. An accuracy over 70% denotes a good classification model and an accuracy over 80% an excellent model. However, accuracy cannot be trusted alone when the used dataset is imbalanced—that is, 1 class of the examined images has very few instances in comparison with other classes. Therefore, in such cases, the calculation of other metrics, such as precision and recall, is very important. Precision is the fraction of samples that were correctly assigned to the positive class by the model to the total number of samples that were assigned to the positive class by the model and recall shows the fraction of samples assigned to the positive class by the model that actually belongs to the positive class. MAP is based on the plot of Precision as a function of Recall. MAP is near 1.0 when the model can correctly handle the prediction of the positive class. F-measure is the harmonic mean of precision and recall. An F-measure equal to 1 indicates perfect precision and recall, while an F-measure equal to zero denotes that either precision or recall is zero. The MAPE is a measure of the accuracy of a prediction model and its formula is given in Table 3, where *A_t_*is the ground truth value and *P_t_* is the predicted value.

**TABLE 3:** Performance evaluation metrics

${\rm{Accuracy}} = \frac{{TP + TN}}{{TP + TN + FP + FN}}$
${\rm{Precision}} = \frac{{TP}}{{TP + FP}}$
${\rm{Recall}} = \frac{{TP}}{{TP + FN}}$
${\rm{F}} - {\rm{measure}} = \frac{2}{{Recal{l^{ - 1}} + Precisio{n^{ - 1}}}}$
${\rm{Mean\,\,Absolute\,\,Percentage\,\,Error}} = \frac{{100}}{{\rm{n}}}\mathop \sum \limits_{{\rm{t}} = 1}^{\rm{n}} \frac{{| {{{\rm{A}}_{\rm{t}}} - {{\rm{P}}_{\rm{t}}}} |}}{{{{\rm{A}}_{\rm{t}}}}}$

### Image-based food-recognition systems using publicly available datasets as input

**Table 4** contains systems that use hand-crafted features and shallow classifiers tested on PAFDs, whereas **Table 5** presents systems that use CNNs in 1 or more steps of an IBFRS tested on PAFDs. **Table 6** shows a collection of the best performances of different studies on the public datasets that are mentioned by more than 1 study in the recent literature.

**TABLE 4:** List of image-based food-recognition systems for dietary assessment based on hand-crafted features and “shallow” classifiers on publicly available food datasets^1^

Reference	User input-preprocessing	Segmentation	Feature extraction	Dimensionality reduction	Classification	Volume estimation	Datasets and performance
(28)	Depth camera		SIFT, LBP, Gabor, and color features		SVM	—	NTU-FOOD ACC = 62.7%
(26)			Bag of Textons, PRICoLBP, and SIFT		Near duplicate image retrieval	—	UNICT-FD889 MAP = 67.5%
(30)		k-Means clustering	SURF, shape, and color features		Borda count scheme	—	Ambient Kitchen Precision = 86.29% Recall = 83.61%
(118)			Superpixel Linear Distance Coding and Locality-constrained Linear Coding, mid-level features		SVM	—	PFID ACC = 50.45%UEC-Food 100 ACC = 60.50%
(66)		Superpixels segmentation	Mid-level food parts approach		SVM	—	UEC-Food 100 ACC = 60.50%
(102)			SIFT, SURF	BOF	SVM	—	UEC-Food 100 ACC = 82.38%
(119)					Metric forests	—	Food-101 ACC = 68.29%
(27)			SIFT, PRICoLBP, Textons, anti-Textons	BOF	ANN with χ^2^distance	—	UNICT-FD1200 ACC = 93.04%
(96)		Wavelet kernel-based Wu-and-Li Index Fuzzy clustering			Whale Levenberg Marquardt ANN	—	UNIMIB 2016 ACC = 96.27%
(99)		Multiple hypothesis segmentation: salient region detection, multi-scale segmentation and fast rejection	Color, texture and local neighborhood pixel features		ANN	—	UNIMIB 2016 ACC = 95.9%
(100)		Canny edge detection, multi-scale segmentation, fast rejection of background pixels	Color, texture, SIFT, and SURF features		3-Layer ANN	—	UNIMIB 2016 ACC = 94.5%
(98)		Local variation segmentation	Color, texture, local descriptors: SIFT and Multi-scale Dense SIFT (MDSIFT)		Multi-kernel SVM	—	UNIMIB 2016 ACC = 93.9%
(33)			Color features, HOG, SIFT, LBP, Locality-constrained Linear Coding	Bag-of-Words	SVM	—	Menu-Match Recall = 83%Calorie estimation Absolute error = 232 ± 7.2
(35)		Plate detection with Hough transform	Color and Edge Directivity Descriptor (CEDD), Gabor features, LBP, Local Color Contrast Chromaticity moments, Complex Wavelet features		KNN	—	UNIMIB 2015 (CEDD) ACC = 99.05%

1 ACC, Accuracy; ANN, artificial neural network; BOF, bag-of-features; HOG, histogram of oriented gradients; KNN, k-nearest neighbors; LBP, local binary patterns; MAP, mean average precision; PFID: Pittsburgh fast-food image dataset; PRICoLBP, pairwise rotation invariant co-occurrence local binary patterns; SIFT, scale-invariant feature transform; SURF, speeded up robust features; SVM, support vector machine.

**TABLE 5:** List of image-based food-recognition systems for dietary assessment based on CNNs on publicly available food datasets^1^

Reference	User input-preprocessing	Segmentation	Feature extraction	Dimensionality reduction	Classification	Volume estimation	Datasets and performance
(32)	—	—	Locality-Constrained Linear Coding, deep features with DeCAF	Bag-of-Words	SVM	—	Dishes (deep features)ACC = 72.88%
(6)	Drawing polygons	Manual segmentation using polygons	CNN	—	CNN	—	Food segmentation: UNIMIB 2016 Recall = 71.4%, Precision = 73.4%, F-measure = 72.4% Food classification: UNIMIB 2016 ACC = 78%
(17)	—	Food localization using CNN	CNN	—	CNN	—	Food segmentation:UEC-Food 256 Precision = 54.33% Recall = 50.86% Egocentric Food Precision = 17.38% Recall = 8.72% Food classification:UEC-Food 256 ACC = 63.16% Egocentric Food ACC = 90.90%
(76)	—	—	CNN	—	SVM	—	PFID ACC = 70.13%
(48)	Top-view photo including credit card	Color-pixel-based k-means clustering and GrabCut		—	CNN	Based on the size of the reference object	UEC-Food 100 ACC = 75%
(88)	—	—	—	—	CNN	—	Food-101 ACC = 88.28%UEC-Food 100 ACC = 81.45%UEC-Food 256 ACC = 76.17%
(120)	—	—	CNN including semantics-aware features	—	CNN	—	Food-101 ACC = 72.11%
(37)	—	—	CNN exploiting the joint relation between food and ingredient labels through multi-task learning	—	CNN	—	Food classification:VIREO Food-172 ACC = 82.06%UEC-Food 100 ACC = 82.12%Ingredients recognition:VIREO Food-172 F-measure = 67.17%UEC-Food 100 F-measure = 70.72%
(77)	—	—	CNN	—	CNN	—	UEC-Food 100 ACC = 60.9%
(121)	—	—	Covariances of convolutional layer feature maps of CNN	—	CNN	—	Food-101 ACC = 58.65%
(122)	—	—	CNN	—	CNN	—	UEC-Food100 ACC = 76.3%Food-101 ACC = 77.4%
(123)	—	—	Feature vector from ensemble of 3 CNNs	—	CNN	—	Food-101 ACC = 71.12%
(124)	—	Food border defined by user with a circle	CNN	—	CNN	—	FooDD ACC = 94.11%
(125)	—	—	CNN	—	Multi-Task Triplet Network	—	UEC-Food 256 MAP = 31.7%
(110)	—	—	CNN	—	CNN (NutriNet)	—	UNIMIB 2016 ACC = 86.39%
(126)	—	—	CNN	—	CNN	—	Food-101 ACC = 87.96%UEC-Food 100 ACC = 86.51%UEC-Food 256 ACC = 78.60%
(127)	—		CNN	—	CNN	—	Food-101 ACC = 86.97%
(92)	—	—	CNN (DualNet)	—	Ensemble of CNNs	—	UEC-Food 100 ACC = 49.19%
(39)	—	—	CNN	—	SVM	—	NTUA-Food 2017 ACC = 85.94%
(128)	—	—	CNN	—	CNN (Inception V3)	—	Food-101 ACC = 81.65%
(103)		CNN (Mask R-CNN)	CNN (ResNet50)	—	CNN	CNN (VolumeNet)	Food segmentation:MAP = 64.7%Food classification:Madima 2017 ACC = 93.33%
(129)	—	—	CNN	—	CNN (WISeR)	—	UEC-Food 100 ACC = 89.58%UEC-Food 256 ACC = 83.15%Food-101 ACC = 90.27%
(130)	—	—	—	—	CNN	—	UNIMIB 2016 ACC = 77.5%
(22)	—	—	—	—	CNN (VGG, ResNet-50, Wide ResNet-50, Inception V3)	—	Food classification:ChinaFood-100 ACC = 78.26% (Inception V3)Nutrients estimation:Protein, fiber, vitamin, calcium, and ironMAPE is approximately 65%
(36)	—	—	CNN (VGG)	—	SVM	—	Instagram800K ACC = 72.8%
(40)	Top view image and side view image, a coin as a fiducial marker	GrabCut and Faster R-CNN	CNN	—	CNN	—	ECUST Food Dataset Mean Error = ±20%
(41)	—	—	CNN (ResNet)	—	CNN (ResNet)	—	Food524DB ACC = 69.52%
(45)	—	—	Multi-task CNN (ResNet)	—	CNN (ResNet)	—	Eating Occasion Image to Food Energy ACC = 88.67% MAE = 56.82 (kcal)
(46)	—	Faster R-CNN provides bounding boxes with a foodness score	CNN (DenseNet-121)	—	Multi-task CNN (DenseNet-121)	—	Food localization:UEC-Food 100 Precision = 82%, Recall = 86%, F-Measure = 84%UEC-Food 256 Precision = 94%, Recall = 88%, F-Measure = 91%VIPERFoodNet Precision = 79%, Recall = 64%, F-Measure = 71%Food classification:Food-101 ACC = 80%UPMC Food-101 ACC = 69%UEC-Food 100 ACC = 81%UEC-Food 256 ACC = 72%VIPERFoodNet ACC = 72%
(22)	—	—	—	—	CNN (VGG, ResNet-50, Wide ResNet-50, Inception V3)	—	Food classification:ChinaFood-100 ACC = 78.26% (Inception V3)Nutrients estimation:Protein, fiber, vitamin, calcium, and ironMAPE is approximately 65%
(36)	—	—	CNN (VGG)	—	SVM	—	Instagram800K ACC = 72.8%
(40)	Top view image and side view image, a coin as a fiducial marker	GrabCut and Faster R-CNN	CNN	—	CNN	—	ECUST Food Dataset Mean Error = ±20%
(41)	—	—	CNN (ResNet)	—	CNN (ResNet)	—	Food524DB ACC = 69.52%
(45)	—	—	Multi-task CNN (ResNet)	—	CNN (ResNet)	—	Eating Occasion Image to Food Energy ACC = 88.67% MAE = 56.82 (kcal)
(46)	—	Faster R-CNN provides bounding boxes with a foodness score	CNN (DenseNet-121)	—	Multi-task CNN (DenseNet-121)	—	Food localization:UEC-Food 100 Precision = 82%, Recall = 86%, F-Measure = 84%UEC-Food 256 Precision = 94%, Recall = 88%, F-Measure = 91%VIPERFoodNet Precision = 79%, Recall = 64%, F-Measure = 71%Food classification:Food-101 ACC = 80%UPMC Food-101 ACC = 69%UEC-Food 100 ACC = 81%UEC-Food 256 ACC = 72%VIPERFoodNet ACC = 72%
(22)	—	—	—	—	CNN (VGG, ResNet-50, Wide ResNet-50, Inception V3)	—	Food classification:ChinaFood-100 ACC = 78.26% (Inception V3)Nutrients estimation:Protein, fiber, vitamin, calcium, and ironMAPE is approximately 65%
(36)	—	—	CNN (VGG)	—	SVM	—	Instagram800K ACC = 72.8%
(40)	Top view image and side view image, a coin as a fiducial marker	GrabCut and Faster R-CNN	CNN	—	CNN	—	ECUST Food Dataset Mean Error = ±20%
(41)	—	—	CNN (ResNet)	—	CNN (ResNet)	—	Food524DB ACC = 69.52%
(45)	—	—	Multi-task CNN (ResNet)	—	CNN (ResNet)	—	Eating Occasion Image to Food Energy ACC = 88.67% MAE = 56.82 (kcal)
(46)	—	Faster R-CNN provides bounding boxes with a foodness score	CNN (DenseNet-121)	—	Multi-task CNN (DenseNet-121)	—	Food localization:UEC-Food 100 Precision = 82%, Recall = 86%, F-Measure = 84%UEC-Food 256 Precision = 94%, Recall = 88%, F-Measure = 91%VIPERFoodNet Precision = 79%, Recall = 64%, F-Measure = 71%Food classification:Food-101 ACC = 80%UPMC Food-101 ACC = 69%UEC-Food 100 ACC = 81%UEC-Food 256 ACC = 72%VIPERFoodNet ACC = 72%
(93)	—	—	—	—	Ensemble of CNNs (VGG16, VGG19, GoogLeNet, ResNet, Inception V3) with 5 different combination rules (minimum, average, median, maximum, product)	—	Food-101 ACC = 84.28%UEC-Food 100 ACC = 84.52%UEC-Food 256 ACC = 77.20%
(131)	—	—	Category and ingredient oriented feature extraction based on CNN (VGG-16); fusion of 2 different kinds of features	—	Adapted CNN	—	Food-101 ACC = 55.3%VIREO Food-172 ACC = 75.1%ChineseFoodNet ACC = 66.1%
(97)	Two meal images from 2 different viewing angles, 90 and 75 degrees from the table's plane, or short video, fiducial marker	Automated segmentation based on Mask-RCNN. Semi-automatic segmentation based on region growing and merging algorithm	—	—	CNN (Inception V3)	—	Madima database segmentation results: Fmin = 83.9%, Fsum = 94.4%Madima database food recognition results ACC = 57.1%
(99)	—	Multiple hypothesis segmentation: salient region detection, multi-scale segmentation and fast rejection	Color, texture, and local neighborhood pixel features	—	ANN	—	UNIMIB 2016 ACC = 95.9%
(117)	—	—	—	—	Multi-relational graph convolutional network, termed mRGCN (ResNet-50)	—	VIREO Food-172 ACC = 24.2% of unseen ingredientsUEC-Food 100 ACC = 17.9% of unseen ingredients
(116)	—	—	First approach with Bag-of-Words extracts texture (binary patterns), color, SURF, and geometry features	First approach Bag-of-Words	First approach ANN; second approach CNNs such as GoogLeNet, Inception-v3, ResNet101	—	16 Categories were selected from UEC-Food 256. ACC = 93% (ResNet)
(132)	—	Faster R-CNN provides bounding boxes (maximum 5 per image)	—	—	CNN (VGGNet)	—	UEC-Food 100 MAP = 17.5%UEC-Food 256 MAP = 10.5%.
(38)	—	—	—	—	CNN (GoogLeNet)	—	FOOD-5K ACC = 99.2%Food-11 ACC = 83.6%.
(133)	—	—	First approach: feature extraction from AlexNet and VGG16	—	First approach: SVM; second approach: fine-tuning CNN (ResNet50)	—	First approach: FOOD-5K ACC = 99.00%Food-11 ACC = 89.33%Food-101 ACC = 62.44%.Second approach:Food-101 ACC = 79.86%
(134)	—	—	—	—	5-Layer CNN	—	UEC-Food 100 ACC = 60.90%
(100)	—	Canny edge detection, multi-scale segmentation, fast rejection of background pixels	Color, texture, SIFT, and SURF features	—	3-Layer ANN	—	UNIMIB 2016 ACC = 94.5%
(135)	Data augmentation by translations, rotations, shearing, zooming, and flipping	—	—	—	CNN (Inception-v3)	Ingredients and nutritional value estimation from vector space embeddings of words (text data from the internet)	Food-101 ACC = 80.0%
(98)	—	Local variation segmentation	Color descriptors: SCD, DCD. Texture descriptors: EFD and GFD. Local descriptors: SIFT and MDSIFT.	—	Multi-kernel SVM	—	UNIMIB 2016 ACC = 93.9%
(86)	—	—	Pretrained CNNs (GoogLeNet and ResNet152)	—	NB, SVM-RBF, SVM-Poly, ANN, RF	—	FOOD-5K (ResNet-152 and SVM-RBF) ACC = 98.8%Food-11 (ResNet-152 and ANN) ACC = 91.34%Food-101 (ResNet-152 and SVM-RBF) ACC = 64.98%
(136)	—	Weakly supervised CNN model with a new pooling technique and incorporate a class activation map for graph-based segmentation (VGG-16)	—	—	CNN (VGG-16)	—	Food-101 ACC = 74.02%
(137)	—	JSEG algorithm consists of color quantization and spatial segmentation	—	—	—	—	UNIMIB 2016F-measure = 58%

1 ACC, accuracy; ANN, artificial neural network; CNN, convolutional neural network; DCD, dominant color descriptor; EFD, entropy-based categorization and fractal dimension estimation; GFD, Gabor-based image decomposition and fractal dimension estimation; MAP, mean average precision; MAPE, mean absolute percentage error; MDSIFT, multi-scale dense SIFT; NB, naive Bayes; PFID, Pittsburgh fast-food image dataset; Poly, polynomial; RBF, radial basis function; RF, random forest; SCD, scalable color descriptor; SIFT, scale-invariant feature transform; SURF, speeded up robust features; SVM, support vector machine.

**TABLE 6:** Comparison of the performance of different image-based food-recognition systems on publicly available food datasets^1^

Dataset	Reference	CNN-based approach	ACC, %	Mean Average Precision, %
PFID	(118)	–	50.45	—
	(76)	+	70.13	
UEC-Food 100	(66)	–	60.5	
	(48)	+	75	
	(88)	+	81.45	
	(37)	+	82.12	
	(77)	+	60.9	
	(122)	+	76.3	
	(102)	–	82.38	
	(118)	–	60.50	
	(126)	+	86.51	
	(92)	+	49.19	
	(129)	+	89.58	
	(46)	+	81.0	
	(93)	+	84.52	
	(117)	+	17.9	
	(132)	+	—	17.5
	(134)	+	60.90	
UEC-Food 256	(17)	+	63.16	
	(88)	+	76.17	
	(125)	+	—	31.7
	(126)	+	78.60	
	(129)	+	83.15	
	(46)	+	72.0	
	(93)	+	77.20	
	(116)	+	93.0	
	(132)	+	—	10.5
Food-101	(88)	+	88.28	
	(120)	+	72.11	
	(121)	+	58.65	
	(122)	+	77.4	
	(123)	+	71.12	
	(126)	+	87.96	
	(127)	+	86.97	
	(119)	–	68.29	
	(128)	+	81.65	
	(129)	+	90.27	
	(46)	+	80.0	
	(93)	+	84.28	
	(131)	+	55.3	
	(133)	+	79.86	
	(135)	+	80.0	
	(86)	+	64.98	
	(136)	+	74.02	
UNIMIB 2016	(6)	+	78.0	
	(99)	–	95.9	
	(100)	–	94.5	
	(98)	–	93.9	
	(110)	+	86.39	
	(96)	–	96.27	
	(130)	+	77.5	
VIREO Food-172	(37)	+	82.06	
	(131)	+	75.1	
	(117)	+	24.2	
Madima 2017	(103)	+	93.33	
	(97)	+	57.1	
FOOD-5K	(133)	+	99.0	
	(86)	+	98.8	
	(38)	+	99.2	
Food-11	(133)	+	89.33	
	(86)	+	91.34	
	(38)	+	83.6	

1 ACC, accuracy; CNN, convolutional neural network; PFID, Pittsburgh Fast-food Image Dataset; –, not using a CNN-based approach; +, using a CNN-based approach.

Among the 59 studies that used public datasets as input to the IBFRS for dietary assessment, 14 (24%) studies used hand-crafted features and “shallow” classifiers and 45 (76%) studies used CNNs in 1 of the phases of the IBFRS. Among these 59 studies, 17 (29%) used the Food-101; 16 (27%) the UEC-Food 100; 11 (19%) the UNIMIB 2016; 9 (15%) the UEC-Food 256; 3 (5%) the FOOD-5K, the Food-11, and the VIREO Food-172; 2 (3%) the PFID and the Madima 2017; and 1 (2%) the UNICT-FD1200, the EgocentricFood, the FooDD, the NTUA-Food 2017, the ChinaFood-100, the VIPERFoodNet, the UPMC Food-101, the ChineseFoodNet, the NTU-FOOD, the UNICT-FD889, the Ambient Kitchen, the Dishes, the Menu-Match, the UNIMIB-2015, the Instagram 800 K, the ECUST Food, the Food524DB, and the Eating Occasion Image to Food Energy public dataset.

Among the 14 studies that used hand-crafted features and “shallow” classifiers on public datasets, 7 (50%) studies used Scale-Invariant Feature Transform (SIFT) features as input to the “shallow” classifiers. Color features were also frequently used (7 studies; 50%). Dimensionality reduction was implemented in 3 (21%) of these studies with the BOF and the Bag-Of-Words methods. For classification purposes, ANN (4 studies; 29%) and SVM (6 studies; 43%) were the most frequently adopted “shallow” classifiers. Among these studies, a modified ANN achieved the best classification results (ACC = 96.27%) on the UNIMIB 2016 public dataset (96) and an SVM achieved the best classification performance (ACC = 82.38%) on the UEC-Food 100 public dataset (102).

Among the 45 studies that used CNNs in 1 of the phases of the IBFRS, 3 (7%) of them used fiducial markers to aid the photometric calibration and the volume estimation phase of the system. Seven (16%) studies used CNNs also in the segmentation or food localization phase. Other seldomly used methods were manual segmentation, clustering, and canny edge detection. Among these 45 studies, 28 (62%) used CNNs for feature extraction, since CNNs can extract very rich image descriptors. On the other hand, only 4 studies (9%) used hand-crafted color and texture descriptors in the feature extraction phase. Dimensionality reduction was not adopted, with the exception of 2 studies (4%) that used the Bag-Of-Words method. Classification was implemented with CNNs in 35 (78%) studies. CNNs were also used for the final volume estimation phase in 1 study (2%) (103).

CNNs outperformed shallow classifiers combined with hand-crafted features in 8 out of 9 public datasets (PFID, UEC-Food 100, UEC-Food 256, Food-101, VIREO Food-172, Madima 2017, FOOD-5K, and Food-11) that were used in more than 1 study as input to the IBFRS (Table 6) due to their ability to conceive complex spatial relations of pixels in images. The only exception was the classification performance of a modified Whale Levenberg Marquardt ANN on the UNIMIB 2016 (96), which outperformed all other “shallow” and deep approaches. This inferior performance of the CNNs on the UNIMIB 2016 dataset can be attributed to the small number of images (1027) of this dataset in comparison to the other public datasets (e.g., UEC-Food 100 contains 10,000 images and Food-101 contains 101,000 images).

### Image-based food-recognition systems supporting dietetic professional practice

Going beyond the systematic review, we searched the literature to find supportive evidence for the benefits of the application of IBFRS in dietetic professional practice. During the last decade over 10,000 mobile phone applications have been developed for weight management and diet monitoring (104). Since mobile devices, such as mobile phones and tablets, started carrying a camera, it was soon realized that they could be used towards improving dietary assessment and monitoring. Initially, mobile applications used static images and there is important evidence that they ameliorated dietetic practice. For example, researchers used photos of meals and snacks taken with mobile phones to encourage children to increase their fruit and vegetable consumption (105). In this way, by using mobile applications that capture photos of the consumed meals, dietitians can monitor children's nutrition against obesity and related chronic diseases, such as hypertension and impaired glucose tolerance. In another study, the challenges of diet monitoring of adolescents with intellectual and developmental disabilities were confronted by prompting them to use a mobile device to take photos of all food and beverages they consumed over a specified period (106). Photo-assisted records improved the overall estimates of energy and macronutrient intake compared with food records completed by proxies (parents). The latest advances in the field of mobile applications for weight management and diet monitoring concern the embedding of IBFRS relying on computer vision approaches. Examples of such diet-related mobile applications that embed an IBFRS module for supporting dietetic professional practice are shown in **Table 7**. Based on the available data, these applications are used by thousands up to millions of users.

**TABLE 7:** Popular diet-monitoring or weight-management mobile applications that embed an image-based food-recognition system module

Mobile application name	Downloads, *n*
LoseIt	>10,000,000
MyNetDiary	>1,000,000
Foodvisor	>500,000
Bitesnap	>100,000
Calorie Mama AI	>100,000
Ate Food Journal	>100,000
See How You Eat	>100,000
MealLogger	>50,000

As mentioned above, a foremost domain of the application of IBFRS is diet monitoring for improving the management of chronic diseases, such as diabetes. According to the Agency for Healthcare Research and Quality, several mobile applications for diabetes self-management were associated with improvement in important biomarkers, such as glycated hemoglobin (HbA1c) (107). Vasiloglou et al. implemented an IBFRS, GoCARB, and compared it with 6 experienced dieticians in terms of estimating the carbohydrate intake content of individuals with diabetes (108). It was found that the IBFRS estimated the carbohydrate content with the same accuracy as the professional nutritionists. In this way, the IBFRS GoCARB may be an invaluable tool towards diabetes self-management by offering individuals with diabetes the option of an easy-to-use, accurate, and almost real-time estimation of the carbohydate content of their plated meals (108). In another study, a mobile nutritional management program integrated into the web-based program, Diabetes Mellitus Dietary Management Guide (DMDMG), for individuals with diabetes was implemented by Ahn et al. (109). The program was evaluated in terms of nutrition knowledge, dietary attitude, eating behavior, and diet intake with individuals with diabetes who used the system and non-users for 1 mo. The study results showed that the program users showed increased healthful dietary behavior. In addition, more users had higher nutrition knowledge scores after the 1-mo trial than non-users. Moreover, dietary intake of calcium and sodium significantly increased in the non-user group, while the user group did not show significant changes. The results of this study show that the program had created positive changes in patients’ dietary life (109).

Geriatrics is another area where IBFRS can play an important role by aiding the dietary assessment of elderly patients. A dataset of self-acquired images from individuals with Parkinson disease taken with a mobile phone camera was collected, and the CNN using this dataset as input achieved a good accuracy, encouraging the implementation of mobile applications using real-world images (110).

IBFRS can also be used in the hospital setting to measure patients’ food consumption and to inform the dietitian if patients’ nutritional needs are adequately met (111). For example, an application was developed that gave the ability to the patients to capture with a mobile device their initial food serving and their leftovers (112). The images were then sent to the hospital server and were analyzed by dietitians towards food intake estimation.

Diet-related mobile apps are also used by sports dietitians for supporting athletes for better health and athletic performance. According to the study by Jospe et al. (113), 32.4% of sports dietitians who participated in the study used mobile diet-related apps to help athletes assess and track their dietary intake. One of the most frequently used mobile apps by sports dietitians in this study was “Lose It,” which has an IBFRS module. The participating sports dietitians stated that the mobile diet-related apps were very or somewhat effective in assisting them to assess their clients’ diet or even to assist their clients to assess their own diet (113). Costello and colleagues (114) guided elite adolescent athletes to record their energy intake via an estimated food diary and the application “Snap-n-Send,” which incorporates an IBFRS, combined with a 24-h dietary recall interview. Their dietary intake was fully provided and weighed by the research team in advance. The results of this study were promising since they show the ability of the mobile application “Snap-n-Send” to accurately determine the dietary intake of the athletes (114). Simpson and colleagues (115) presented the feasibility of the mobile app, MealLogger, embedding an IBFRS module, to increase knowledge and promote healthy nutritional behaviors within a group of elite athletes. During this study, participants reported a highly positive experience of application use. They reported positive changes in dietary behaviors based on in-app education. All participants preferred this method to traditional methods of dietary analysis (115).

## Discussion

Diet-monitoring systems can help the experts (e.g., medical professionals, nutritionists) and the individual to understand his/her eating habits and behavior, and therefore improve his physical condition, while reducing the risk of the development of diet-related diseases.

Manual record-keeping and recall methods, such as food records, 24-h dietary recall, and FFQs, and mobile applications, such as MyFitnessPal, have been proposed by the experts for food-intake monitoring, since they are simple to follow and costless (5). However, they are tedious and individuals often fail to be accurate and to comply with such tools for a long period of time. On the other hand, automatic record-keeping approaches, such as methods that use a camera of the user's mobile device, seem to simplify the process. In particular, methods that combine a camera on the user's mobile device with computer vision and machine-learning techniques are very easy to use by individuals and more objective, since they do not rely on the ability of the individual to remember or assess the macronutrient content of the consumed meal. Moreover, research studies have shown that dietary monitoring of individuals with diabetes and the elderly might improve by using IBFRS (108–110). In addition, other research studies have shown that athletes’ dietary monitoring and nutrition education were improved by using such IBFRS (113–115). Moreover, in the study by Furtado (116), when the performance of a human reviewer was compared with the performance of an automatic food-recognition approach based on CNNs, it was proven that the human reviewer was inferior to the automatic approach when the food items that were presented were unknown to him/her previously (i.e., before training for that survey).

Limitations of these systems might be attributed to the lack of appropriate input datasets. Thus, for the optimization of these automated systems’ performance, appropriate food datasets need to be publicly available. Two large PAFDs, Food-101 and VIREO Food-172, consist of fast-food and Chinese food only, respectively (37). Moreover, until now, less than 10 different cuisines have been included in the existing PAFDs. Thus, the need for the creation of more novel datasets from cuisines from all over the world is obvious. It is also important that some of the novel datasets should be large enough for the training of CNNs. In addition, there is a need for food datasets related to the diet of people suffering from metabolic diseases, such as diabetes or metabolic syndrome, originating from different countries. Our analysis of IBFRS also shows the emerging need for creating future public food datasets that contain additional information for food images, such as ingredients, nutrients, sodium content, or cooking methods, to enable better tracking of dietary goals of individuals and chronic disease prevention and management. It is also obvious that it is very important for accurate dietary assessment that more datasets, apart from UNIMIB 2015, should be created that also contain both initial and leftovers images of meals.

To tackle the lack of datasets from local cuisines, another approach could also be adopted, as proposed by Chen et al. (117), where unknown food items can be recognized with respect to previously known food items. In Chen et al. (117), graph CNNs were trained to recognize previously unseen ingredients by using relations between known and unknown ingredients. Three kinds of relations were adopted: hierarchy (parent–child), attributes (color, shape, cooking method) and co-occurrence.

Observing the best results of the classification accuracy for the PAFDs in Table 6, it can be deduced that they are achieved with the use of CNNs. Although CNNs are demanding in terms of size of training datasets, specifications for CPUs or GPUs, the large number of parameters, and long running times, their performance justifies their expanded use (75). Thus, since CNNs prove to be effective for the segmentation, feature extraction, and classification steps, more applications should focus on their exploitation for the creation of more sophisticated methods.

Existing systems have already shown impressive results, as shown in Tables 4 and 5, but there are still open issues that should be tackled in the near future. A first step for the improvement in current diet-monitoring systems is to take into account additional information apart from the food images, such as the user's dietary history, dietary goals and targets, wellness/illness including medication uptake, allergies, and time of uptake of each meal information, and thus personalize the diet-monitoring application. Diet-monitoring applications could be also expanded in order to give the opportunity to the user to add new personal images or new food categories on the training food image database from the user's daily routine.

Finally, the embedding of more sensors, such as accelerometers and gyroscopes, or other types of cameras, such as thermal and depth cameras, in the next generations of mobile devices could improve the existing diet-monitoring applications in terms of volume estimation.

In conclusion, this systematic review provides an overview of diet-monitoring systems for the reduction in the risk of diet-related chronic diseases. Studies describing dietary assessment systems based on a camera on the user's mobile device combined with computer vision and machine-learning techniques have been thoroughly examined and the methods used as well as the performance achieved are described in the previous sections. Diet-monitoring systems can be broken down into the following phases: image depiction, segmentation, feature extraction, dimensionality reduction, classification, and volume and calories estimation. For the optimization of the systems’ performance, appropriate PAFDs need to be constructed. From evaluation metrics, it can be observed that food-recognition systems have evolved and the segmentation, features extraction, and classification performance have improved by using CNNs. For example, the classification accuracy achieved on the Food-101 dataset increased from 55.3% to 90.27% with the use of CNNs. Several studies show that the professional dietitian and the individual can benefit from such systems in terms of diet monitoring and nutrition self-education. However, despite the progress that has been done, challenges regarding the methods applied and the creation of appropriate public food datasets remain.

## Notes

This research has been co-financed by the European Union and Greek national funds through the Operational Program Competitiveness, Entrepreneurship, and Innovation, under the call Support for Regional Excellence in R&D Infrastructure (MIS 5047289).

Author disclosures: The authors report no conflicts of interest.

Abbreviations used: ANN, artificial neural network; BOF, bag-of-features; CNN, convolutional neural network; FFQ, food-frequency questionnaire; FN, false negative; FP, false positive; GPU, graphics processing unit; HSV, hue-saturation-value; IBFRS, image-based food recognition systems; ILSVRC, ImageNet Large-Scale Visual Recognition Challenge; KNN, k-nearest neighbor; LAB, Lightness AB; LBP, local binary pattern; MAP, mean average precision; MAPE, mean absolute percentage error; PAFD, publicly available food dataset; PFID, Pittsburgh Fast-food Image Dataset; PRICoLBP, pairwise rotation invariant co-occurrence local binary pattern; RF, random forest; RGB, red-green-blue; SVM, support vector machine; TN, true negative; TP, true positive.
